# Mechanically durable tri-composite polyamide 6/hematite nanoparticle/tetra-n-butylammonium bromide (PA6/α-Fe_2_O_3_/TBAB) nanofiber based membranes for phosphate remediation

**DOI:** 10.3389/fchem.2024.1472640

**Published:** 2024-09-09

**Authors:** Yun Young Choi, Dung Thi Hanh To, Sewoon Kim, David M. Cwiertny, Nosang V. Myung

**Affiliations:** ^1^ Department of Chemical and Biomolecular Engineering, University of Notre Dame, Notre Dame, IN, United States; ^2^ Department of Civil and Environmental Engineering, University of Iowa, Iowa City, IA, United States; ^3^ Department of Chemistry, University of Iowa, Iowa City, IA, United States

**Keywords:** electrospinning, nylon, iron oxide, flexible composite nanofibers, phosphate removal

## Abstract

Essential properties for a Point of Use (POU) water filter include maintaining high removal capacity and rate, with excellent mechanical properties to withstand pressure drop. Herein, mechanically robust tri-composite polyamide 6/iron oxide nanoparticles/tetra-n-butylammonium bromide (PA6/α-Fe_2_O_3_/TBAB) nanofiber composite membranes were electrospun for phosphate (P) remediation, where the diameter and composition were tuned by controlling solution compositions and electrospinning conditions. Tri-composite composition and morphology affect phosphate uptake where the adsorption capacity followed Langmuir isotherm whereas the adsorption kinetics followed pseudo second order behavior. Mechanical properties (i.e., Young’s Modulus (*E*) and toughness) were significantly influenced by the composition and morphology of the tri-composite, as well. Although additional TBAB and iron oxide decreased toughness, there are optimum composition ranges which resulted in maximum Young’s Modulus. Of the synthesized nanofiber membranes, PA6/α-Fe_2_O_3_/TBAB nanofibers with 17% α-Fe_2_O_3_ and 2% TBAB showed excellent phosphate uptake capacity [i.e.*,* 8.9 mg/g (52 mg of P/g of α-Fe_2_O_3_)] while it is bendable, stretchable, and able to plastically deform without fracturing (i.e., Young’s modulus of 2.06 × 10^8^ Pa and Toughness of 1.35 × 10^6^ J m^−3^). With concerns over the impact of P on water resources and the long-term availability of limited P resources, this tri-composite membrane is well suited for applications in both wastewater treatment and resource recovery.

## 1 Introduction

Phosphorus (P) is one of the essential nutrients needed for humans, animals, and plants. However, excess phosphorus leaching from various sources including agricultural, industrial, and residential runoff can result in eutrophication (Fang et al., 2017; Dodds et al., 2009). Various techniques such as biological treatment, chemical precipitation, ion exchange, and adsorption have been utilized to remove phosphate from the environment (Zheng et al., 2023). For example, biological treatment relies on polyphosphate accumulating organisms (PAOs) to remediate P from environments through alternating anaerobic and aerobic conditions. Although it is eco-friendly, it requires strict operational conditions to maintain remediation capabilities (Zheng et al., 2023). Chemical precipitation uses metal ions to precipitate phosphate from contaminated water source, which then is removed by settling or filtration process. Compared to others, it requires high operating cost and can lead to large amounts of flocculant and coagulant with sludge treatment (Zhang et al., 2012). Additionally, both techniques cannot reduce phosphate concentration below 0.1 mg P/L which is above EPA recommendation limit of 0.05 mg/L for total phosphates in streams (Clune et al., 2020).

Compared to the other techniques, adsorption is one of most cost-effective, high capacity, efficient, and eco-friendly approaches to remove P to meet EPA recommendation (Cui et al., 2020; Singh et al., 2018). Various adsorbents such as metal oxides, carbonaceous materials, metal organic frameworks (MOFs), and their derivatives have been utilized for P remediation (Priya et al., 2022; Mitrogiannis et al., 2017; Huang et al., 2018; Huang et al., 2010; El Hanache et al., 2019a; El Hanache et al., 2019b; Anjum et al., 2019; Xie et al., 2017; Sonoda et al., 2020; Prashantha Kumar et al., 2018; Meftah and Zerafat, 2016). Among these adsorbents, metal oxides or their derivatives have been popular choices because of their high affinity toward phosphate with low toxicity, low cost, and high pH stability (Sun et al., 2020). Most common metal oxides utilized for P capture are iron oxides, aluminum (hydr)oxides (Yang et al., 2007), lanthanum (Fang et al., 2018) and cerium oxides (Wu and Lo, 2020). Commercially available P adsorbents such as E33 and Phoslock are based on iron oxide and lanthanum incorporated bentonite, respectively.

Iron oxides are one of most popular P adsorbents because of their high affinity, low cost, and earth abundance. Ajmal et al. investigates various iron oxides including goethite (α- Fe(OH)O), magnetite (Fe_3_O_4_) and ferrihydrite (Fe(OH)_3_) with different surfaces toward P capture. They found that P adsorption capacity depended on the surface area and crystal phase (Ajmal et al., 2018). Highest P adsorption capacity per surface area was found in goethite (0.58 mg/m^2^) followed by magnetite (0.47 mg/m^2^) and ferrihydrite (0.372 mg/m^2^). Wang et al. examined for P removal using hydrous iron oxide modified diatomite. When diatomite was modified with hydrous iron oxide, the specific surface area increased 152 times from 0.53 to 80.44 m^2^/g and show approx. 25 times improvement of P adsorption capacity (Wang et al., 2016). Since adsorption is a surface phenomenon, greater surface to volume ratio results in higher adsorption capacity. While metal oxide nanoparticles have a high efficiency of P removal, challenges still exist such as aggregation and need of additional separation and removal steps (Nalbandian et al., 2016; Nalbandian et al., 2022).

Chemical-active composite nanofiber-based membrane eliminates the need for recovery steps with optimized performance and functionality. Compared to conventional adsorbent materials like metal and ceramics, nanofiber-based membrane reinforced composites exhibit distinct features of dual functionality to be used as both filters and adsorbents (Rajak et al., 2019; Rajak et al., 2022). It provides physical separation through the three-dimensional network while multiple active components can be embedded to remediate a wide spectrum of water pollutants (Nalbandian et al., 2022). Although there are other methods, composite nanofibers can be cost efficiently manufactured using scalable electrospinning with controlled composition and morphology via fine tuning of its synthesis conditions (Yu et al., 2022; Tlili and Alkanhal, 2019). Unlike other methods, electrospinning allows for fabrication of composite nanofibers in a single synthesis step which significantly reduces the fabrication time and cost. Due to their uncontrolled fiber orientation, high void fraction and porosity, electrospun nanofiber materials often require enhancement and modification to be applied in practical contexts (Science Direct, 2024).

Previously, we demonstrated the ability to fabricate composite nanofibers using electrospinning (Peter et al., 2016; Hesterberg Butzlaff et al., 2023; Peter et al., 2017; Egodawatte et al., 2016; Kim et al., 2019; Ding et al., 2022; Greenstein et al., 2019; Peter et al., 2018) for various environmental and energy applications. More specifically, quaternary ammonium salt (TBAB) encapsulated hematite nanoparticles (α−Fe_2_O_3_ NPs) embedded polyacrylonitrile (PAN) tri-composite nanofibers were fabricated and utilized for P capture (Wang et al., 2021). Systematic studies indicated that surface enriched (α-Fe_2_O_3_) nanoparticles were primarily responsible for chemical adsorption of phosphates with some additional contribution to uptake from TBAB through anion exchange. Although this work demonstrated that the composite nanofiber membrane with high interconnected porosity can be used to remediate P without the need of sorbent recovery step, one of the remaining barrier to the implement of electrospun membranes for applications in wastewater treatment and resource recovery applications is the development of mechanically durable sheet to meet the demand of water treatment applications (Tlili and Alkanhal, 2019; Tang et al., 2022).‬‬‬‬‬‬‬‬‬‬‬‬

In this work, TBAB encapsulated α−Fe_2_O_3_ NPs embedded PA6 nanofibers with controlled composition and morphology were fabricated using a one pot synthesis electrospinning technique. PA6, also known as Nylon6, was selected over other polymer host matrices because of its high mechanical strength and durability as well as its compatibility with other materials to overcome the decrease in mechanical properties for composite membranes (Yadav, 2018). Previous mechanical properties testing of PAN and PA6 nanofibrous mat showed tensile strength of 6.7 MPa and 34.9 MPa, respectively (Sheng et al., 2016; Molnar et al., 2012). A design of experiment approach was implemented to fabricate composite nanofibers with different composition and morphology. The resulting PA6/α−Fe_2_O_3_/TBAB nanofiber membranes show comparable adsorption kinetics and adsorption capacity with superior mechanical properties relative to PAN/α−Fe_2_O_3_/TBAB composite nanofibers (Wang et al., 2021). Mechanical properties such as Young’s modulus, yield strength, tensile strength, toughness, and strain to fracture were optimized to determine the potential to be used in various treatment applications.

## 2 Experimental

### 2.1 Electrospinning solution preparation and characterization

Polyamide-6, tetra-n-butylammonium bromide (TBAB; ≥98%), and potassium antimony tartrate hydrate (≥99%) were purchased from Sigma-Aldrich whereas trifluoroacetic acid (TFA, 99%), acetone (Ace, ACS reagent grade, 99.5%), potassium dihydrogen phosphate (KH_2_PO_4_; 99.3%), sulfuric acid, and ascorbic acid (99.4%) were purchased from Fisher Scientific. Hematite (99.95%, average diameter of 3 nm APS powder) and ammonium molybdate tetrahydrate (99%) were purchased from Alfa Aesar. All materials were used without further treatment. Electrospinning solutions were prepared by dispersing α−Fe_2_O_3_ NPs in a solvent mixture of acetone (Ace) and trifluoroacetic acid (TFA) 60:40 mol%. Once α−Fe_2_O_3_ NPs were well-dispersed using sonication, PA6 pellets and TBAB (various concentrations: 0, 0.1, 1 wt.%) were added to the solution, which was then sealed for magnetic stirring until homogeneous.

Three solution properties (i.e., viscosity, surface tension and electrical conductivity) were measured. The viscosity was measured using a CPA-40 spindle connected to a Brookfield DV2THB viscometer. The solution viscosity was determined to be independent of the shear rate. Thus, the viscosity was measured at 95% torque at each rotational speed ranging from 0.5 rpm to 200 rpm. An automatic surface tensiometer (Shanghai Fangrui Instrument, QBZY-1) with platinum-coated plate was used to measure the surface tension. Solution electrical conductivity was measured using an electrical conductivity probe from Apera Instruments (Al1311, K = 0.1) connected to EZO conductivity circuit (Atlas Scientific), on Tentacle T3 using Raspberry Pi (Whitebox T3, Mkll). All solution property measurements were taken at room temperature prior to electrospinning to correlate them closely to the resulting nanofiber properties.

### 2.2 Controlling composite nanofiber morphology during electrospinning through design of experiment (DOE)

Electrospinning was conducted by injecting the prepared solution through a 5-mL BD Luer-Lok syringe with a 20-gauge stainless steel needle using a syringe pump (New Era, NE-100). Negative voltage was applied to the needle tip while the drum collector, wrapped with aluminum foil and rotating around 400 rpm, was grounded to collect the sample. Electrospinning and environmental conditions including applied voltage, feed rate, temperature, and absolute humidity were fixed at 12 kV, 0.25 mL/h, 23°C ± 1°C, and 0.008 ± 0.001 kg H_2_O/kg dry air, respectively.

An experimental approach that varies one factor at a time can overlook conditions critical to developing a full systematic understanding of experimental design and fails to examine the interaction between the factors (Yu et al., 2022; Yu and Myung, 2018). DOE allows one to systematically vary factors of different conditions at the same time and fully visualize the effect of each factor on the resulting system properties. From the DOE analysis, an equation can be obtained that allows one to predict the response or conditions to achieve a specific, target response.

The first DOE consists of varying the α−Fe_2_O_3_:PA6 ratio and TBAB concentration while keeping the concentration of PA6 at 6.5 wt.%. The conditions for the α−Fe_2_O_3_:PA6 ratio and TBAB concentration were based on previous work done by Wang et al. (2021) with PAN tri-composites, which had adsorption capacity of 8.76 mg P/g of nanofiber membrane. The first DOE was conducted to study how adding and increasing α−Fe_2_O_3_ NP and TBAB concentration to PA6, a more hydrophilic polymer compared to PAN, would affect solution and nanofiber properties. The lower and upper limit for the α−Fe_2_O_3_ NP:PA6 ratio were chosen to be 0 and 0.43 with middle point set at 0.21 for α−Fe_2_O_3_ NP:PA6 ratio. TBAB concentration was altered from 0 wt.% to 1 wt.%. The middle point for TBAB concentration was set at 0.1 wt.% rather than median to study the effect of TBAB more drastically within a narrow range. These samples are listed as Sample 9, 8, 2, 1, and 3 in Table 1. The code for each sample in Table 1 shows what factors (the α−Fe_2_O_3_ NP:PA6 ratio and TBAB concentration) and the range (the lower, middle, or upper point) have been altered to synthesize each sample.

**TABLE 1:** Effect of electrospinning solution compositions on the solution properties and nanofiber morphology. DOE #1 was designed to study the effect of α-Fe_2_O_3_ and TBAB with a fixed PA6 content, whereas DOE #2 was designed to investigate the effect of PA6 and α-Fe_2_O_3_ content while fixing TBAB content.

S#	Code	Sample name	Electrospinning Solution Composition			Solution Properties		
			PA6 [wt.%]	Fe_2_O_3_ PA6 ratio	TBAB [wt.%]	μ [cP]	γ [dynes/cm]	σ [mS/cm]
Design of Experiment (DOE) #1								
9	(−,−)	1.00 PA6	6.5	0	0	17.13	21.13	1,304
8	(−,+)	0.86PA6_0.00Fe2O3_0.14 TBAB	6.5	0	1	14.52	21.0	1916
2	(0,0)	0.81PA6_0.17Fe2O3_0.02 TBAB	6.5	0.21	0.1	21.45	21.2	1,387
1	(+,−)	0.70PA6_0.30Fe2O3_0.00TBAB	6.5	0.43	0	45.78	21.4	1,279
3	(+,+)	0.63PA6_0.27Fe2O3_0.10 TBAB	6.5	0.43	1	68.8	21.3	1807
Design of Experiment (DOE) #2								
6	(−,−)	0.73PA6_0.16Fe2O3_0.11 TBAB	6.5	0.21	1	86.33	21.6	1714
7	(−,+)	0.55PA6_0.36Fe2O3_0.09 TBAB	6.5	0.65	1	69.72	20.8	1719
4	(0,0)	0.64PA6_0.27Fe2O3_0.09 TBAB	7	0.43	1	63.18	21.3	1,697
5	(+,−)	0.74PA6_0.16Fe2O3_0.1 TBAB	7.5	0.21	1	43.95	21.1	1,667
10	(+,+)	0.56PA6_0.36Fe2O3_0.07 TBAB	7.5	0.65	1	250.1	19.6	1,678

The second DOE was designed and carried out based on the nanofiber properties and batch testing results of the first DOE. To target a bigger range of average fiber diameters, the concentration of PA6 was increased from 6.5 wt.% to 7.5 wt.%. From the first DOE, samples with higher ratio of α−Fe_2_O_3_ NP:PA6 ratio had higher phosphate removal efficiency. To test if increasing the α−Fe_2_O_3_ NP:PA6 ratio further increases the adsorption capacity, the range of α−Fe_2_O_3_ NP:PA6 ratio was increased from 0 to 0.43 (i.e., the range from the first DOE) to 0.21 to 0.65. For second DOE, the TBAB concentration was kept at 1 wt.%; while addition of TBAB helped to decrease the bead density, the first DOE revealed there was less than 2% difference in phosphate removal efficiency between 0.1 wt.% and 1.0 wt.% TBAB. The samples from the second DOE are listed as Sample 6, 7, 4, 5, and 10 in Table 1.

### 2.3 Nanofiber characterization

Morphology of the as-spun nanofiber was observed with a scanning electron microscope (Prisma E SEM, Thermo Fisher Scientific, USA). Prior to analysis, a thin layer of gold was sputtered using Electron Microscopy Sciences 575X over the samples at 20 mA for 30 s to minimize surface charging. Obtained SEM images were imported to ImageJ software to measure the average fiber diameter, which was obtained by measuring the diameter of 30 unique nanofibers. The bead density was calculated by dividing the total number of beads from a single SEM image by the total area of the image. Fiber fraction was determined by the proportion of nanofibers in the total product, which could include beads and clumps. Transmission electron microscopy (TEM) samples were collected by placing a carbon-coated copper grid directly in front of the drum collector for 1 min during electrospinning. TEM images were captured using 300 (S)TEM Ceta™.

The specific surface area and pore volume measurements of the composite nanofibers were obtained using a surface area and pore size analyzer (Quantachrome Nova 4200e) under nitrogen. Specific surface area (S_BET_) was determined from multi-point BET, where the relative pressures (P/P_0_) ranged from 0.05 ≤ P/P0 ≤ 0.30 (Hesterberg Butzlaff et al., 2023). The t-plot (V-t, where V is the volume of gas adsorbed and t is the statistical film thickness) method was applied to the adsorption isotherm to determine the micropore volume and surface area (Kruk et al., 1996; Galarneau et al., 2014; Daraghmeh et al., 2017).

Mechanical properties were examined using a Discovery hybrid rheometer (DHR-30, TA Instruments, USA) attached with RH Linear Tension Rectangular Fixture. Nanofiber samples were placed between the plates between the plates and the samples were pulled apart at a constant linear rate of 1.0 mm per second until 50 mm is reached at room temperature. All measurements were obtained directly from the manufacturer supplied computer software (TRIOS, TA Instruments).

### 2.4 Phosphate adsorption studies

To evaluate the P remediation efficiency of PA6/α-Fe_2_O_3_/TBAB nanofiber membranes, adsorption kinetics and isotherm studies were carried out using 40 mg of composite nanofiber membranes in 40 mL of phosphate solution with phosphate concentrations of 2, 5, 10, 15 and 20 mg/L. The experiment was performed in a 50 mL sealed polypropylene centrifugal tube at 23°C. The solution was collected using a 5-mL BD Luer-Lok syringe with a syringe filter attached (0.22 μm, PTFE Teflon filter) at different time intervals (0.25, 0.5, 1, 2, 4, 8 and 24 h). The phosphate concentration of collected samples was measured at 880 nm with a UV-Vis spectrophotometer (Agilent Cary 60) based on the ascorbic acid molybdate blue method (Wang et al., 2021).

The equilibrium adsorption capacity (q_e_) was calculated using the following equation (Equation 1):

$$q_{e}=\frac{\left(C_{o}-C_{e}\right)*V}{m}$$ (1)

where q_e_ (mg/g) is the adsorption capacity at time t, C_0_ and C_e_ are the initial and equilibrium phosphate concentrations (mg/L), respectively, V is the volume of the solution (L), and *m* is the mass of the composite nanofiber mat (g).

The adsorption kinetic was investigated using the pseudo first order and pseudo second order equations (Equations 2, 3 respectively).

$$q_{t}=q_{e}*\left(1-\exp\;\left(k_{1}*t\right)\right)$$ (2)

$$\frac{t}{q_{t}}=\frac{1}{\left(k_{2}q_{e}^{2}\right)+\frac{t}{q_{e}}}$$ (3)

where rate constant of pseudo first order adsorption as k_1_ (min^−1^) and k_2_ as rate constant of pseudo second order adsorption (g mg^−1^ min^−1^); q_e_ is the amount of phosphate adsorption at equilibrium (mg/g); q_t_ is the amount of phosphate adsorption at time t (min) in mg/g. Additionally, the adsorption isotherms were investigated using Langmuir and Freundlich adsorption equations (Equations 4, 5 respectively):

$$q_{e}=\frac{\left(q_{\max}K_{L}C_{e}\right)}{\left(1+K_{L}C_{e}\right)}$$ (4)

$$q_{e}=K_{F}C_{e}^{1/n}$$ (5)

where q_max_ is the maximum adsorption capacity (mg/g); C_e_ is the equilibrium phosphate concentration (mg/L); *n* is the parameter of the Freundlich adsorption isotherm; and K_L_ (L/mg) and K_F_ ((mg/g)*(L/mg)^1/n^) are the equilibrium constants related to the Langmuir and Freundlich adsorption isotherms, respectively.

## 3 Results and discussion

### 3.1 Effect of solution conditions on solution properties

Table 1 lists the solution properties and nanofiber morphologies from DOE #1 and #2. The analysis for the DOE #1 of viscosity, electrical conductivity, and surface tension as function of α−Fe_2_O_3_ NP:PA6 ratio and TBAB concentrations are shown in Supplementary Figure S1. The viscosity of the solution drastically increased from 17.13 cP to 57.29 cP as α−Fe_2_O_3_ was added and the ratio of α−Fe_2_O_3_ NP:PA6 was increased to 0.43 without adding TBAB to the solution. The addition of TBAB to solution resulted in a decrease in the viscosity from 17.13 cP to 14.52 cP. The decrease in viscosity is attributed to increase in free charge in polymer phenomenon aligns with observations reported in previous literatures (Mingzheng et al., 2012; Zheng et al., 2014). However, when 1 wt.% of TBAB was added to the electrospinning solution with α−Fe_2_O_3_ NP:PA6 ratio, the viscosity of the solution increased. Although both factors lead to increase in viscosity of the electrospinning solution, increase in α−Fe_2_O_3_ NP:PA6 ratio had more significant impact on the viscosity. Based on these results, the equation shown in Supplementary Figure S1A can be used to predict the solution viscosity when the α−Fe_2_O_3_ NP:PA6 ratio and TBAB concentration are varied.

The electrical conductivity of the solution reflects the charge density on a jet, and the elongation level of a jet by an electrical force (Wendorff et al., 2012; Alves et al., 2013; Ko and Wan, 2014). It was reported in previous literature that an increase in electrical conductivity can result in a thinner fiber diameter of electrospun polymer fibers (Xue et al., 2019; Sun et al., 2014). Low electrical conductivity leads to more beads, resulting from insufficient elongation of a jet by electrical force needed to produce uniform nanofibers (Cooper et al., 2018; Wang et al., 2014; Bhardwaj and Kundu, 2010). The electrical conductivity of the solution was mainly affected by TBAB concentration. Supplementary Figure S1B shows that increasing α−Fe_2_O_3_ NP:PA6 ratio from 0 to 0.43 decreased the electrical conductivity, which may be explained by increased amount of α−Fe_2_O_3_, which has poor conductivity. Adding TBAB to the solution sharply increased the electrical conductivity from 1,292 μS/cm to 1862 μS/cm, similar to the trend we previously observed for PAN/Fe_2_O_3_/TBAB composite solutions (Wang et al., 2021).

Surface tension plays a critical role in the electrospinning process and determines electrospinnability and determines the upper and lower boundaries of the electrospinning window (Haghi and Akbari, 2007). A Taylor cone is produced when a certain threshold voltage exceeds the value of surface tension (Xue et al., 2019). As shown in Supplementary Figure S1C, the surface tension of the as-prepared solution was generally maintained throughout at 21 dynes/cm. While previous literature showed that incorporation of TBAB to the solution decreases the surface tension for other polymers and/or solvents (as expected for a surfactant), notably that was not the case for this solution (Tanvir and Qiao, 2012).

Analysis for DOE #2, where the concentration of PA6 and α−Fe_2_O_3_ NP:PA6 ratio was varied, can be seen in Supplementary Figure S2. Changing these factors had a bigger impact on the viscosities of the solutions. As the PA6 concentration increased from 6.5 wt.% to 7.5 wt.%, the viscosity of the solution increased, as expected. Chisca et al., reported that an increase in the polymer concentration reduces polymeric chain mobility in solution due to chain entanglement that prevents chain re-ordering and raises the solution viscosity (Chisca et al., 2012). When the α−Fe_2_O_3_ NP:PA6 ratio was increased from 0.21 to 0.65, a similar trend that seen in DOE #1 was observed, and the viscosity of the solution increased from 65.14 cP to 159.91 cP. Although both factors had a direct effect on the viscosity, when the concentration of PA6 and the ratio of α−Fe_2_O_3_ NP:PA6 both increased, the combination of factors had the biggest impact on increasing the solution viscosity, which is shown as the steepest slope shown in Supplementary Figure S2A and through the equation describing this relationship (given above the graph) as well. The electrical conductivity of the solution decreased from 1717 μS/cm to 1,673 μS/cm when the PA6 concentration increased. Unlike the DOE #1, when the α−Fe_2_O_3_ NP:PA6 ratio increased, the electrical conductivity increased slightly.

### 3.2 Effect of solution conditions on nanofiber morphology and dimension

**FIGURE 1:**
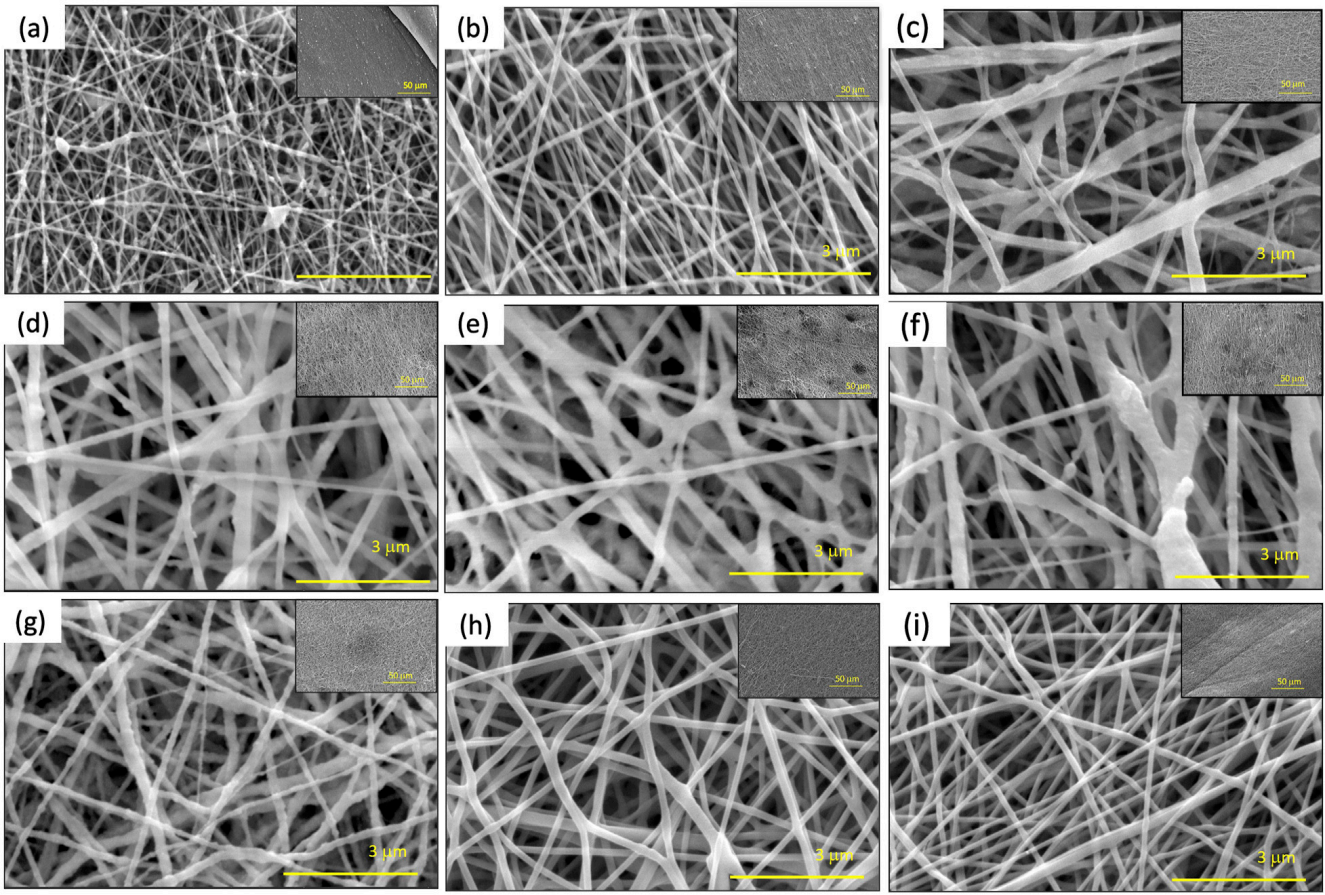
SEM images of polyamide 6/α-Fe_2_O_3_/TBAB tri-composite nanofibers. **(A–I)** corresponds to Sample #1 to Sample #9 respectively.

Figure 1 shows the morphology of electrospun tri-composite nanofibers. As shown in Supplementary Figure S5, tri-composite nanofibers with different composition and fiber diameters ranging from 66 nm to 235 nm were fabricated. For Figures 1A–G, the nanofibers show texture from different amounts of α−Fe_2_O_3_ NP that have been added to the solution, while Figures 1H, I shows smooth electrospun nanofiber without any texture as the solution did not include α−Fe_2_O_3_ NP. The rough surfaces appearing in SEM images indicate that α−Fe_2_O_3_ NP were enriched on the surface of the nanofiber. This was attributed to the interaction between surfactant and α−Fe_2_O_3_ NP leading to improved dispersion of α−Fe_2_O_3_ NP.

#### 3.2.1 Effect of PA6 concentration and α−Fe_2_O_3_ NP:PA6 ratio

To study the effects of how solution properties affect the nanofiber properties such as average fiber diameter and the fiber fraction, a total of 10 solutions were electrospun with the same electrospinning and environmental conditions (Table 1). The DOE analysis in Supplementary Figure S3A shows that increasing the α−Fe_2_O_3_ NP:PA6 ratio decreased the average fiber diameter from 149 nm to 108 nm. As TBAB was added to the solution, there was an increase in the fiber diameter. For fiber fraction, increasing α−Fe_2_O_3_ NP:PA6 ratio resulted in lower fiber fraction and more defects such as beads and clumps (Figure 1). With 1 wt.% TBAB added to the solution, the bead density decreased, and fiber fraction increased. Supplementary Figure S3B shows that when increasing α−Fe_2_O_3_ NP:PA6 6 ratio, adding 1 wt.% of TBAB to the solution can lead to an increase in fiber fraction.

Sample #10 which was composed of 7.5 wt.% PA6, 0.65 for α−Fe_2_O_3_ NP:PA6 ratio and 1 wt.% of TBAB resulted in electrospraying instead of electrospinning. When the α−Fe_2_O_3_ NP:PA6 ratio is kept the same, increasing the PA6 concentration from 6.5 wt.% to 7 wt.% resulted in an increase in average fiber diameter from 160 nm to 218 nm. This is expected since increasing the solution concentration increases the solution viscosity and the average fiber diameter is directly correlated to solution viscosity (Yu and Myung, 2018; Ratanavaraporn et al., 2010; Deitzel et al., 2001). Also, within the same concentration, increasing α−Fe_2_O_3_ NP:PA6 ratio from 0.21 to 0.65 leads to an increase in average fiber diameter from 160 nm to 196 nm. Table 2 shows that increasing the PA6 concentration had a bigger impact on the average fiber diameter compared to increasing the α−Fe_2_O_3_ NP:PA6 ratio. Supplementary Figure S3B shows that the fiber fraction decreases as PA6 concentration and α−Fe_2_O_3_ NP:PA6 ratio increased. This analysis was consistent for DOE #2 as shown in Supplementary Figure S4B of decrease in the fiber fraction as the α−Fe_2_O_3_ NP:PA6 ratio increased, and there were no nanofiber formations for Sample #10 when constant electrospinning and environmental conditions were maintained. However, even with adjustment of electrospinning and environmental conditions, the high ratio of α−Fe_2_O_3_ NP:PA6 may contribute to aggregation of α−Fe_2_O_3_ NP that may, in turn, lead to clogging of the needle and electrospraying rather than electrospinning (Yadav, 2018; Liu et al., 2016; Jiang et al., 2017).

**TABLE 2:** Morphology, surface area, and adsorption volume of synthesized nanofibers.

S #	Code	Sample name	Ave. fiber dia. [nm]	Fiber Fraction [μm^2/^μm^2^]	Bead density [beads/μm^2^]	Surface area [m^2^/g]	Single point adsorption total pore volume of pores [cm^3^/g]	t-Plot micropore volume [cm^3^/g]
9	(−,−)	1.00 PA6	130 ± 25	0.996	0.004	5.21	6.88E-03	2.65E-04
8	(−,+)	0.86PA6_0.00Fe2O3_0.14 TBAB	165 ± 29	0.993	0.007	13.82	2.17E-02	1.77E-03
2	(0,0)	0.81PA6_0.17Fe2O3_0.02 TBAB	118 ± 30	0.962	0.038	10.5	1.58E-02	8.41E-04
1	(+,−)	0.70PA6_0.30Fe2O3_0.00TBAB	66 ± 18	0.978	0.022	4.34	6.21E-03	2.23E-04
3	(+,+)	0.63PA6_0.27Fe2O3_0.10 TBAB	149 ± 69	0.989	0.011	15.84	2.75E-02	9.29E-04
6	(−,−)	0.73PA6_0.16Fe2O3_0.11 TBAB	160 ± 51	0.983	0.017	8.97	1.55E-02	−7.11E-04
7	(−,+)	0.55PA6_0.36Fe2O3_0.09 TBAB	195 ± 76	0.984	0.016	9.22	1.64E-02	−1.68E-04
4	(0,0)	0.64PA6_0.27Fe2O3_0.09 TBAB	235 ± 61	0.984	0.016	0.63	2.50E-05	N/A
5	(+,−)	0.74PA6_0.16Fe2O3_0.1 TBAB	218 ± 66	0.974	0.026	4.27	3.45E-03	1.53E-03
10	(+,+)	0.56PA6_0.36Fe2O3_0.07 TBAB	—	—	—	—	—	—

#### 3.2.2 Effect of TBAB content

To investigate the effects of surfactant on resulting solution and nanofiber properties, tetrabutylammonium bromide (TBAB) was added to the solution. Incorporation of TBAB in the solution decreased the surface tension and increase the viscosity as discussed in Section 3.1. Comparing Sample #1 and Sample #3 from SEM images shown in Figure 1, both samples have fixed α−Fe_2_O_3_ NP:PA6 ratio of 0.43, however with addition of TBAB, it is shown that there are less beads present in Sample #3. The rest of the samples show a similar trend of lower bead density compared to Sample #1, which does not have TBAB, while the rest of the samples had 1 wt.% of TBAB except Sample #2, which had 0.1 wt.% of TBAB mixed into the solution. Similar findings have been reported in the literature, where adding a small concentration of surfactant increased the viscosity of the solution and decreased the surface tension, allowing for smooth and increased fiber fraction for pristine nanofibers (Yu and Myung, 2018; Lin et al., 2004). Lee et al. suggested that bead formation during electrospinning can occurs in a low viscosity polymer solution, and Jun et al. reported that addition of an organic salt to the solution can lead to reduction of bead formation (Lee et al., 2003; Zeng et al., 2003). During electrospinning, the jet of nanofibers ejected under an electric field exhibit whipping instability, which mainly depends on the charge repulsion overcoming the surface tension. Usually, beads are a product of the instability of the jet under the electric field, thus lowering the surface tension and increasing the charge density suppresses bead formation (Tang et al., 2022; Park et al., 2008). Ultimately, the addition of TBAB helps with lowering bead density and increasing the fiber fraction of the composite nanofibers.

#### 3.2.3 High resolution transmission electron microscope (HR-TEM) with elemental mapping

**FIGURE 2:**
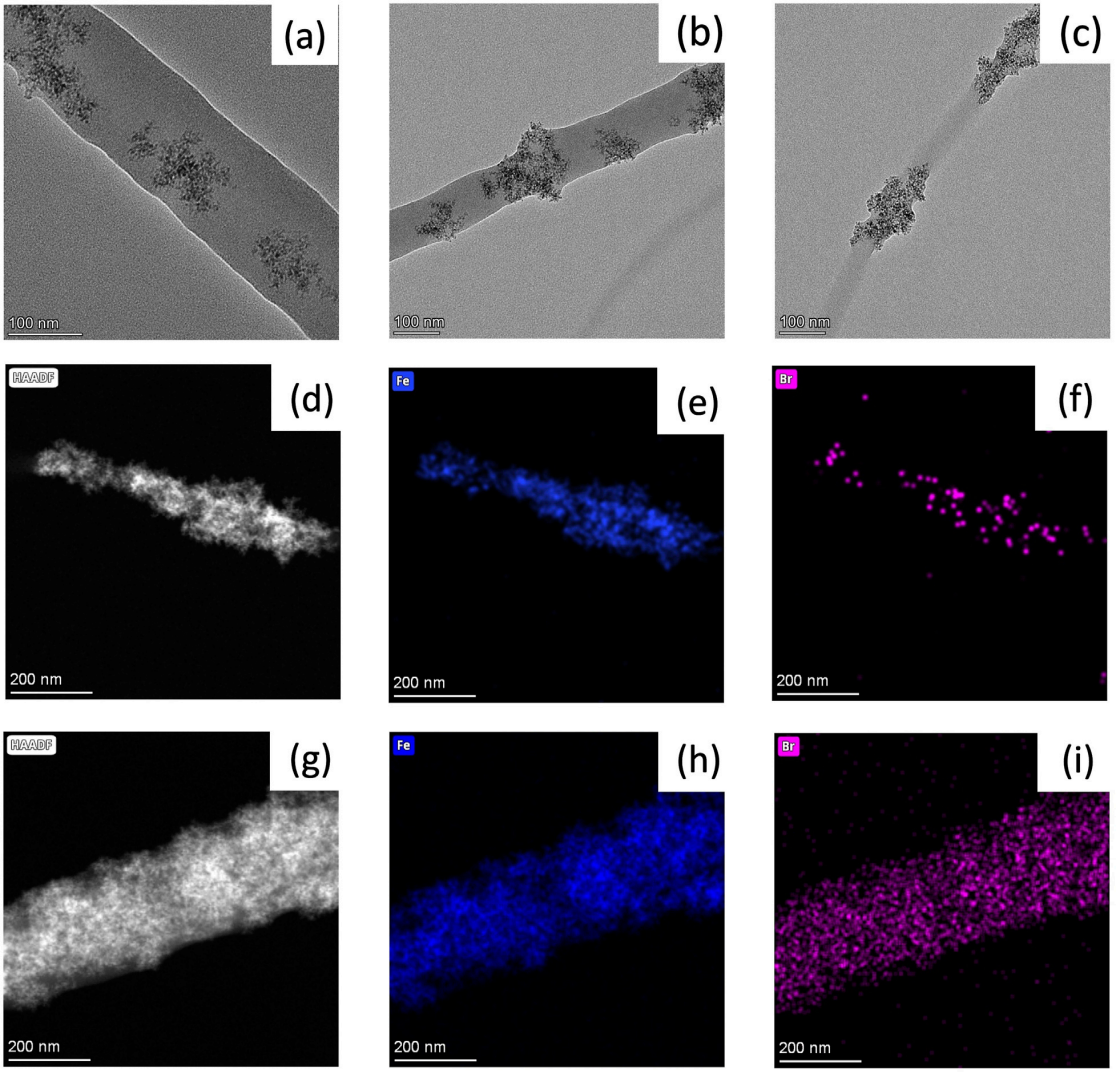
TEM images and mapping of composite nanofibers. **(A)** S#1, **(B)** S#2, **(C)** S#6; **(D)** HAADF image, **(E)** iron, and **(F)** bromide mapping of S#3 and **(G)** HAADF image, **(H)** iron, and **(I)** bromide mapping of S#7.

HR-TEM images of Sample #1, #2, #6 were taken to determine α−Fe_2_O_3_ NP distribution among the nanofibers (Figure 2). Sample #1, which does not contains TBAB shows that α-Fe_2_O_3_ are embedded within the nanofiber (Figure 2A). Unlike Sample #1, Sample #2 shows that most of α−Fe_2_O_3_ NP were located on the edges of nanofiber (Figure 2B). When a TBAB concentration of 1.0 wt.% is reached (Sample #6), α−Fe_2_O_3_ NPs are clearly seen on the edges of the nanofiber (Figure 2C). As shown in our prior works, the presence of TBAB helps to enrich α−Fe_2_O_3_ NPs to the surface of nanofibers. In addition to HR-TEM, elemental mapping of nanofibers was conducted to further understand the distribution of α−Fe_2_O_3_ NPs and TBAB within nanofiber. Figures 2D, G show high-angle annular dark field (HAADF) images of Sample #3 and #7, respectively. Figures 2E, H are an iron elemental mapping whereas Figures 2F, I show the bromide elemental mapping. As shown in the figures, bromide from TBAB shows overlapped dispersion with α−Fe_2_O_3_ NPs, rather than being dispersed evenly within the nanofiber.

#### 3.2.4 Surface area

**FIGURE 3:**
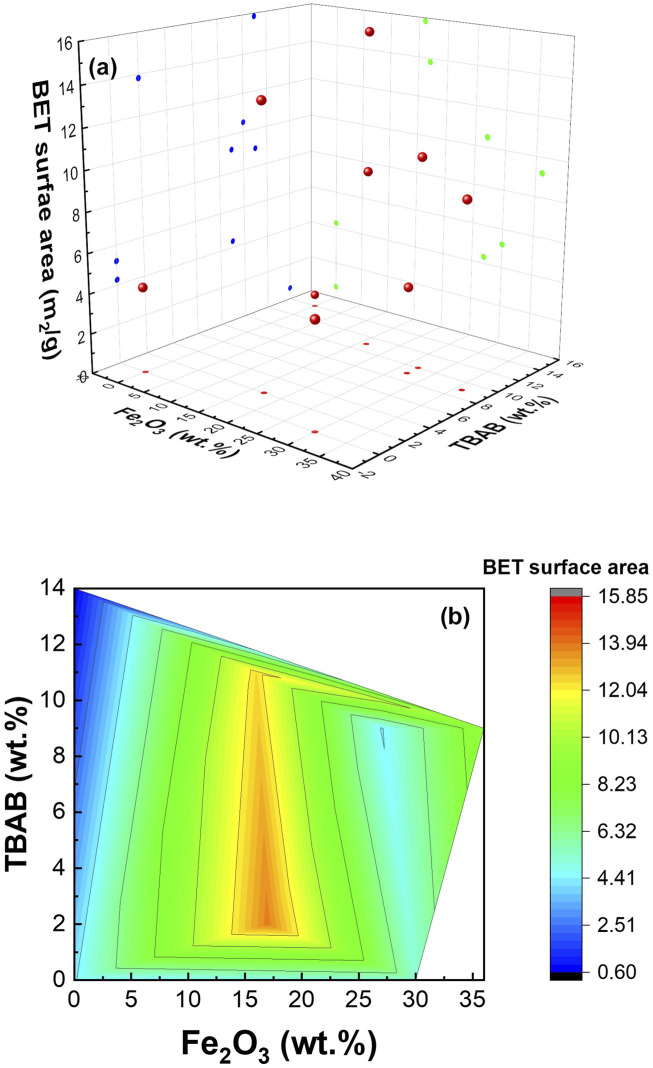
3D **(A)** and contour plot **(B)** of BET surface area as a function of composition.

BET analysis was conducted to determine the surface area of various samples. As expected, the available surface area was significantly altered by the composition and morphology of the nanofibers. Figure 3 shows the surface area as a function of α−Fe_2_O_3_ NP and TBAB content in 3D (Figure 3A) and contour (Figure 3B) plots. As shown in the figures, the surface area strongly depended on α−Fe_2_O_3_ NP, and the highest surface area (i.e., 15.84 m^2^/g) was observed at 16% α-Fe_2_O_3_ (Sample #5) with high TBAB. The next highest surface (i.e., 13.82 m^2^/g) was observed from Sample #2, which has similar α−Fe_2_O_3_ content but lower TBAB concentration. In the absence of TBAB, adding α−Fe_2_O_3_ NPs resulted in higher surface area. The obtained values for surface area can be found in Supplementary Table S1. Total pore volume (v_tot_) increased with the increase in surface area which is expected and is consistent with previous literatures (Plerdsranoy et al., 2017; Xu et al., 2015).

### 3.3 Phosphate removal batch testing

Before conducting detailed adsorption kinetic and isotherm studies, samples were exposed to a fixed phosphate concentration of 10 mg/L (Supplementary Figure S6). Two additional samples, which are α−Fe_2_O_3_ NPs (Sample #11) and TBAB coated α−Fe_2_O_3_ NPs (Sample #12), have been added as control to determine maximum adsorption capacity of pure materials. As shown in the figure, free floating TBAB coated α−Fe_2_O_3_ NPs show the highest extent of phosphate removal (i.e., 90.35%), whereas pristine PA nanofiber (Sample #9) shows the lowest degree of phosphate removal (i.e., 4.68%). Adding 1 wt.% TBAB to PA6 nanofibers (Sample #8) showed slightly higher phosphate removal (i.e., 16.05%) than pristine PA6 nanofiber, which might be attributed to ion exchange characterized by electrostatic attraction between positively charged quaternary ammonium sites (QAS) and oxyanions (i.e., phosphate) (Nalbandian et al., 2022).Initially, α−Fe_2_O_3_ NPs: PA6 ratio was increased to test for increased phosphate uptake, however, Sample #12 showed that both TBAB and α−Fe_2_O_3_ NPs contributed in uptake of phosphate. Increased α−Fe_2_O_3_ NPs content did not necessarily lead to increased phosphate uptake, and Supplementary Figure S6 shows that there exists an optimal ratio of 8.5:1 between α−Fe_2_O_3_ NPs and TBAB that lead to maximum phosphate uptake of the tri-composite nanofiber mat.

### 3.4 Adsorption kinetics

**FIGURE 4:**
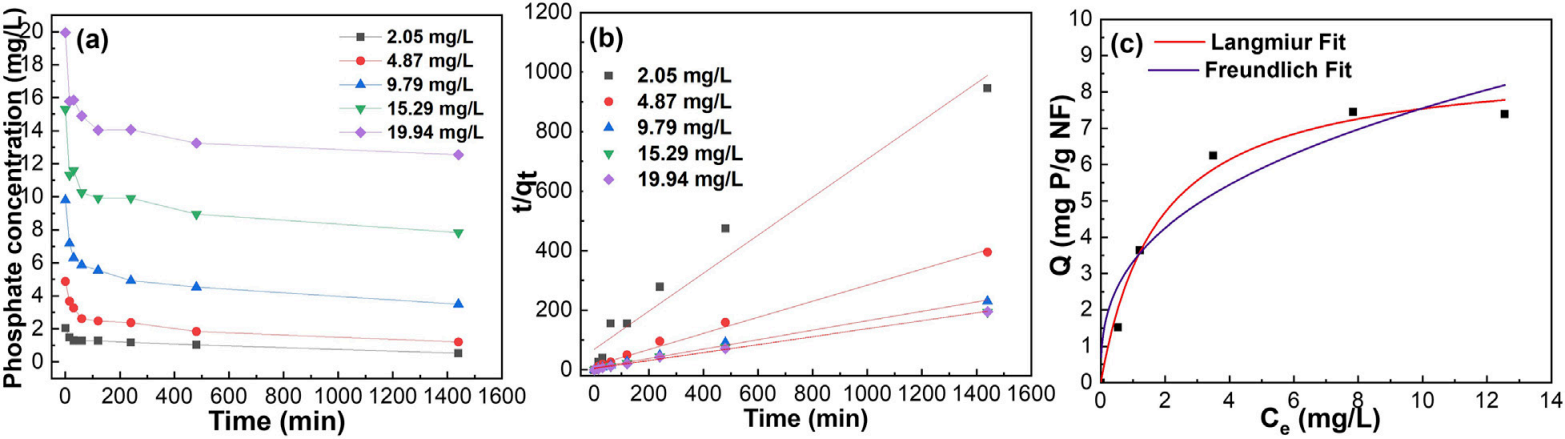
Phosphate removal batch testing using Sample #2: **(A)** raw data, **(B)** adsorption kinetics fitting, **(C)** adsorption isotherm fitting with various concentrations.

Using a batch testing system for phosphate removal from the solution helps to establish the time until equilibrium and rate of phosphate removal. Figure 4A shows the phosphate removal batch testing with different initial phosphate concentrations (i.e., 2, 5, 10, 15 and 20 mg/L). The two most popular adsorption kinetic models, the pseudo first order and pseudo second order were used to fit data. Figure 4B, Supplementary Figures S7, S8 show pseudo first order and pseudo second order fittings of data of PA6/α-Fe_2_O_3_/TBAB nanofiber phosphate removal at different initial phosphate concentrations. Supplementary Table S2 shows summarized kinetic correlation coefficients from the data fitted by pseudo first order and pseudo second order kinetic models. Based on the resulting correlation coefficients (i.e., R^2^ values), it is concluded that PA6/α-Fe_2_O_3_/TBAB nanofiber is best-described by a pseudo second-order kinetic model, which suggests chemisorption via ligand exchange of negatively charged phosphate ions with iron oxide surface (Suresh Kumar et al., 2017). Pseudo-second order fitting has been used to calculate the k_2_, which shows the rate of phosphate adsorption by each of the materials with different contents of α-Fe_2_O_3_ and TBAB. Supplementary Figure S9 shows kinetic rate constant changes as a function of α-Fe_2_O_3_:PA6 ratio. It is observed that while keeping the TBAB concentration constant at 1 wt.%, increasing the α-Fe_2_O_3_ content resulted in a monotonic decrease in k_2_. We interpret this behavior based upon pristine PA6 nanofiber having a higher k_2_ value of 0.38 g/(mg min) than α-Fe_2_O_3_ NP, which only exhibits a value of 0.00386 g/(mg min) (see Supplementary Table S2). Therefore, by adding α-Fe_2_O_3_ into PA6, it is expected that the reaction rate constant will decrease as α-Fe_2_O_3_ content increases.

Meanwhile, increasing the TBAB concentration from 0 to 1 wt.% contributes to a higher adsorption rate constant of 0.00437 g/(mg min) rather than 0.00716 g/(mg min) while the α−Fe_2_O_3_ NP:PA6 ratio remained constant (i.e., 0.43) As discussed previously, incorporating a surfactant such as TBAB to α-Fe_2_O_3_ results in surface enrichment of α-Fe_2_O_3_ nanoparticle in the composite, where the phosphates will be adsorbed by the ligand-exchange reaction with the positively charged surface that may possibly increase the reaction rate (Wang et al., 2021; Jung et al., 2019).

### 3.5 Adsorption isotherms

**FIGURE 5:**
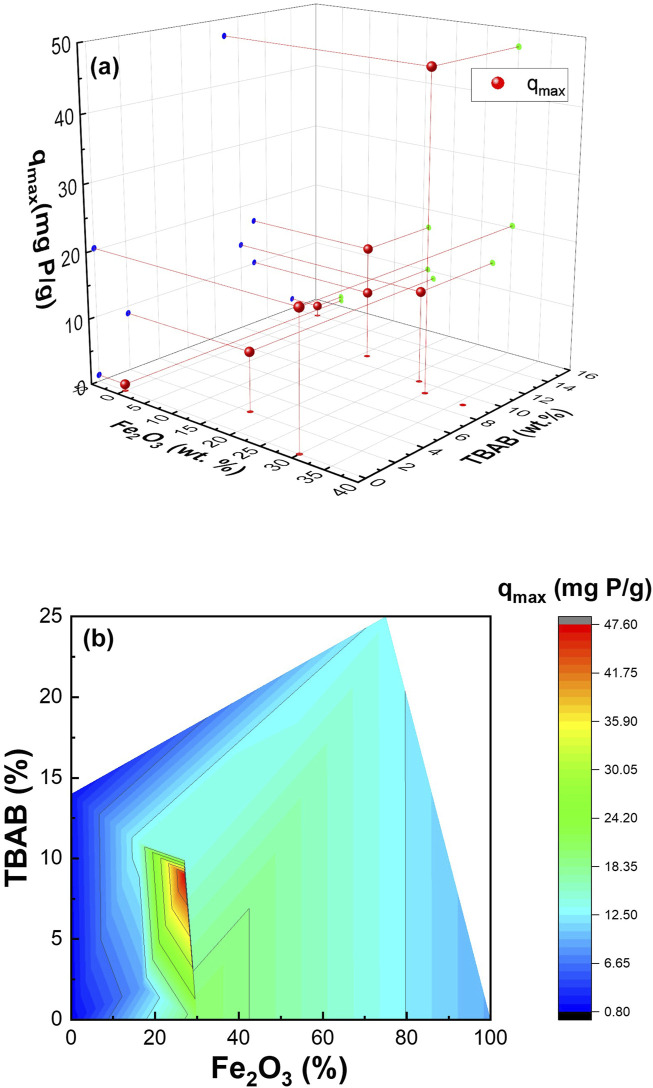
3D **(A)** and contour plot **(B)** of adsorption capacity function of TBAB and α-Fe_2_O_3_ content.

To further understand the adsorption capacity of these composite nanofibers, nonlinear and linear Langmuir and Freundlich isotherm models were used to fit the experimental data. Figure 4C, Supplementary Figures S10, S11 show nonlinear fit to Langmuir and Freundlich model, linear fit to Langmuir model, and linear fit to Freundlich model, respectively. The Langmuir isotherm model describes homogenous, monolayer adsorption onto the finite adsorptive sites whereas the Freundlich isotherm is based on multilayer adsorption on heterogenous sites (Wang et al., 2021; Li et al., 2016; Katal et al., 2012). Supplementary Table S3 summarizes the obtained data, demonstrating that the synthesized composite nanofiber exhibits result comparable to previous finding presented in Supplementary Table S4. Monolayer adsorption onto the active sites was determined by the higher correlation coefficient (R^2^) obtained for Langmuir fitting compared to Freundlich fitting. While the experiment was carried out for all the samples, Sample #7 and Sample #10 failed to be fitted using both Langmuir and Freundlich isotherm equations, which may be because of their high α-Fe_2_O_3_: PA6 ratio. The free-floating particles (as mentioned above) of Sample #11 had q_max_ of 13.22 mg/g of nanoparticles. Figure 5 shows the 3D and contour plot of adsorption capacity as a function of TBAB and α-Fe_2_O_3_ content. The contour plot shows that the adsorption capacity increases as TBAB and α-Fe_2_O_3_ content increases. However, it can be concluded that adsorption capacity of composite nanofiber is more dependent on the α-Fe_2_O_3_ content rather than the TBAB content. As illustrated in Figure 5, an optimal ratio between α-Fe_2_O_3_ and TBAB content is observed, corresponding to optimized adsorption capacity.

### 3.6 Mechanical properties

**FIGURE 6:**
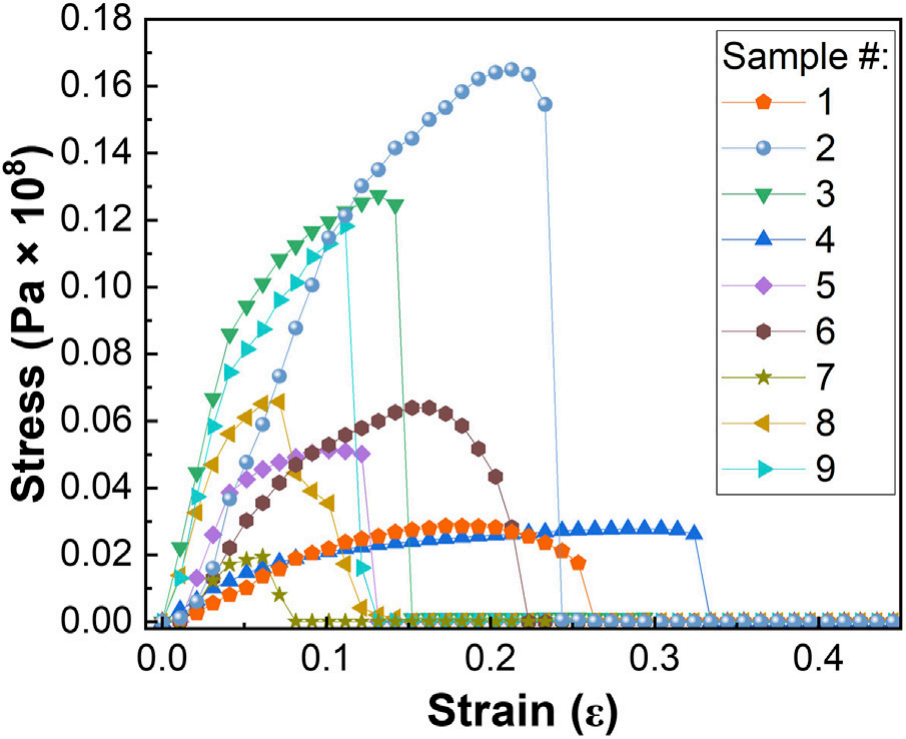
Stress vs. strain graph used to calculate various mechanical properties of PA6/a-Fe_2_O_3_/TBAB nanofibers.

One of the key requirements for a water filtration system is having excellent mechanical properties to withstand high pressure and flux during application (de-Bashan and Bashan, 2004). To understand the impact of adding chemically active ingredients to pristine PA6 nanofibers, composite materials were investigated with tensile strength testing. Figure 6 shows the stress versus strain curves of different samples. Various parameters such as Young’s modulus, yield strength, ultimate tensile strength, toughness, and strain to fracture extracted from the stress-strain plot and the results obtained are listed in Supplementary Table S5. Results showed that in the absence of α-Fe_2_O_3_ NPs, addition of TBAB reduced most of the mechanical properties of PA6 nanofibers. Similar results were observed when TBAB concentration increased from 0.1 wt% to 1.0 wt% while maintaining α-Fe_2_O_3_ content, where the Young’s modulus, yield strength, ultimate tensile strength and toughness decreased but the yield point increased. With an increase in TBAB and α-Fe_2_O_3_ content, the nanofiber became more brittle. Although this is affected by both factors, the slope of the contour plot in Supplementary Figure S12 shows that TBAB content has a predominant effect on the decrease in toughness of the material. Supplementary Figure S13 shows that when there are no α-Fe_2_O_3_ nanoparticles present, increasing the content of TBAB resulted in a decrease in Young’s modulus. Furthermore, when there is no TBAB present, increasing α-Fe_2_O_3_ content also led to a decrease in Young’s modulus; however, when both TBAB and α-Fe_2_O_3_ are added to produce tri-composite nanofibers, Young’s modulus reaches its maximum when α-Fe_2_O_3_ content is between 10%–20% and TBAB content is 2%–10% of the composite nanofibers. The decrease in the mechanical properties with increased amount of α-Fe_2_O_3_ may be due to the inhomogeneous dispersion and aggregation of Fe_2_O_3_ nanoparticles (Yadav, 2018; Liu et al., 2016). The slope of the contour plot of Supplementary Figure S13 shows that while the Young’s modulus is dependent on both TBAB and α-Fe_2_O_3_, it is more dependent on the content of α-Fe_2_O_3_.

Various results were shown in previous literatures in regards of addition of TBAB, such as increasing the tensile strength until 0.66 w/v % but decreasing the tensile strength when TBAB content increases further (Fan et al., 2020). Arora et al. reported that up to 2 wt% TBAB modified clay showed an increase in tensile modulus and tensile strength, but these values decreased when TBAB content was further increased (Arora et al., 2011). In addition, changing the polymeric host results in different mechanical properties. PAN/Fe_2_O_3_/TBAB tri-composite nanofibers have been synthesized by Wang et al. in our previous work (Wang et al., 2021). For their sample with the highest adsorption capacity for phosphate, Young’s modulus, yield strength, ultimate tensile strength, toughness, and strain to fracture was determined to be 6.13 × 10^6^ Pa, 2.30 × 10^5^ Pa, 2.96 × 10^5^ Pa, 1.46 × 10^4^ J×m^−3^, and 7.10 × 10^−2^ ε, respectively. Compared to this work utilizing polyamide 6 instead of PAN, the respective mechanical properties are measured to be 20.51, 16.76, 17.33, 32.33, and 0.27 times higher with PA6, as depicted in Supplementary Table S6. Indeed, it has been reported that the Young’s modulus and tensile strength of nanofibers can depend on several variables such as the chemical structure of polymer, molecular orientation, fiber diameter, fiber fraction, as well as alignment of nanofibers (Lasenko et al., 2023; Pelipenko et al., 2013).

## 4 Conclusion

PA6/α-Fe_2_O_3_/TBAB tri-composite nanofiber membrane was designed and successfully prepared using a one-pot electrospinning synthesis method. Utilizing the design of experiments, the concentration of PA6, Fe_2_O_3_:PA6 ratio, and TBAB concentration have been varied to investigate their effect on the average nanofiber diameter, fiber fraction and bead density. The SEM and TEM images showed TBAB promotes surface enriched α-Fe_2_O_3_ NPs on the nanofibers. The adsorption of phosphate onto the tri-composite nanofibers followed the pseudo second order kinetic model and could be described well using the Langmuir adsorption. The adsorption capacity increased with increasing α-Fe_2_O_3_ content and the kinetic rate constant for phosphate sorption increased with high TBAB. The mechanical properties of composite nanofibers showed an increase in toughness, yield strength, ultimate strength with decreasing α-Fe_2_O_3_ and TBAB content, while Young’s modulus increased with an increase in α-Fe_2_O_3_ content. Considering both adsorptive performance and material properties, the formulation for Sample # 2 was best; it exhibited an adsorption capacity of 8.89 mg/g (52.30 mg of P/g of α-Fe_2_O_3_) while maintaining excellent mechanical properties. Relatively to materials in our prior work, PA6 tri-composite showed 1.4 times higher adsorption capacity as well as 20.51 times higher Young’s modulus, as well as better mechanical properties overall, compared to previously investigated tri-composite nanofibers using PAN as the base polymer.

## Data Availability

The original contributions presented in the study are included in the article/Supplementary Material, further inquiries can be directed to the corresponding author.
