# Supplementary material for: Mechanically durable tri-composite polyamide 6/hematite nanoparticle/tetra-n-butylammonium bromide (PA6/α-Fe2O3/TBAB) nanofiber based membranes for phosphate remediation
Source: Front Chem. 2024 Sep 9;12:1472640. doi: 10.3389/fchem.2024.1472640 (PMC11416959; doi:10.3389/fchem.2024.1472640)
Supplement: Supplementary file 1 [file DataSheet1.PDF]

Supplementary Material

Mechanically Durable Tri-composite Polyamide 6/ Hematite Nanoparticle/ Tetra-n-butylammonium Bromide (PA6/α-Fe_2O_3/TBAB) Nanofiber based Membranes for Phosphate Remediation

Yun Young Choi [1], Dung Thi Hanh To[1], Sewoon Kim[2], David M. Cwiertny[2,3],

Nosang V. Myung [1*]

[1] Department of Chemical and Biomolecular Engineering, University of Notre Dame, Notre Dame, IN 46530, USA. *E-mail:* nmyung@nd.edu

[2] Department of Civil and Environmental Engineering, University of Iowa, 4105 Seamans Center, Iowa City, IA 52242, USA.

[3] Department of Chemistry, University of Iowa, E331 Chemistry Building, Iowa City, Iowa 52242-1294, USA.

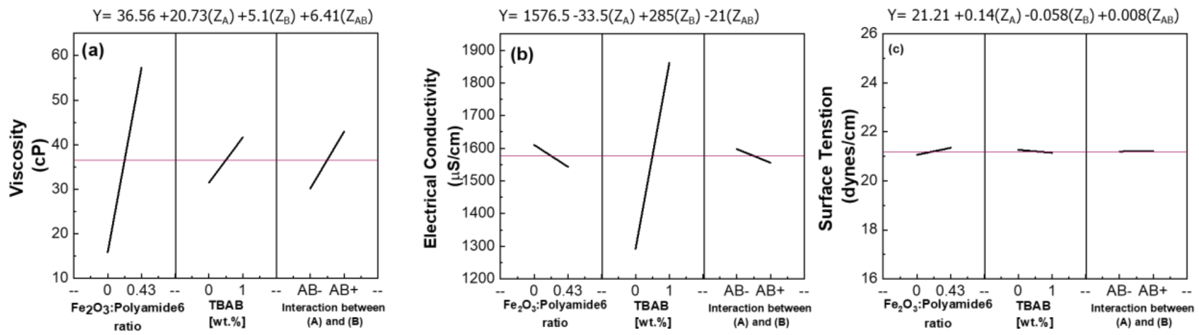

Figure S1. DOE analysis of (a) viscosity, (b) electrical conductivity, and (c) surface tension as a function of solution compositions from first DOE.

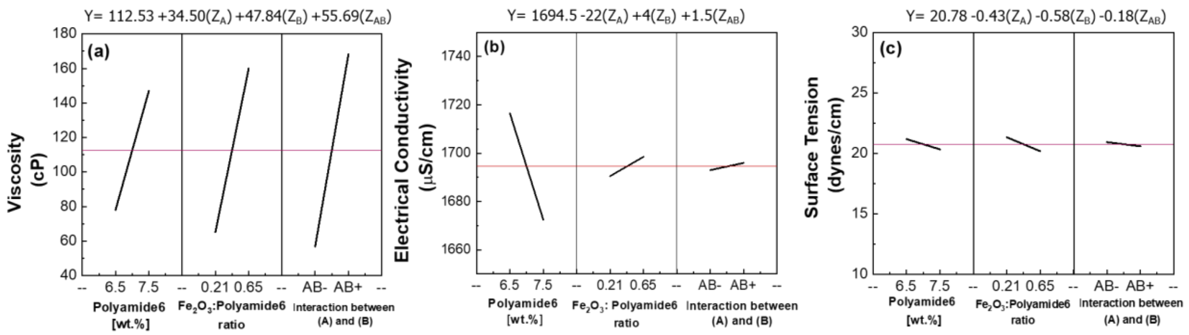

Figure S2. DOE analysis of (a) viscosity, (b) electrical conductivity, and (c) surface tension as a function of solution compositions from second DOE.

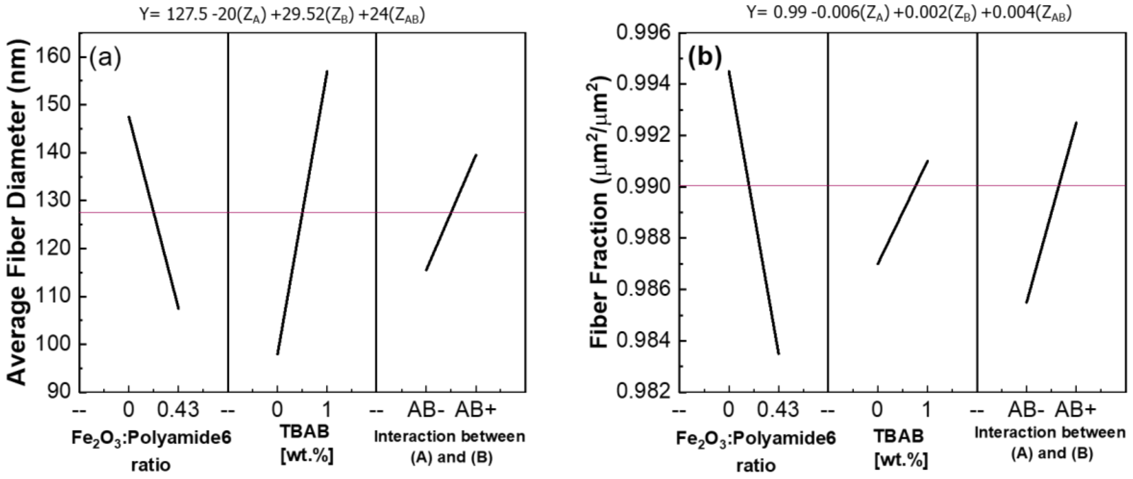

Figure S3. DOE analysis of (a) average fiber diameter and (b) fiber fraction as a function of solution compositions from first DOE.

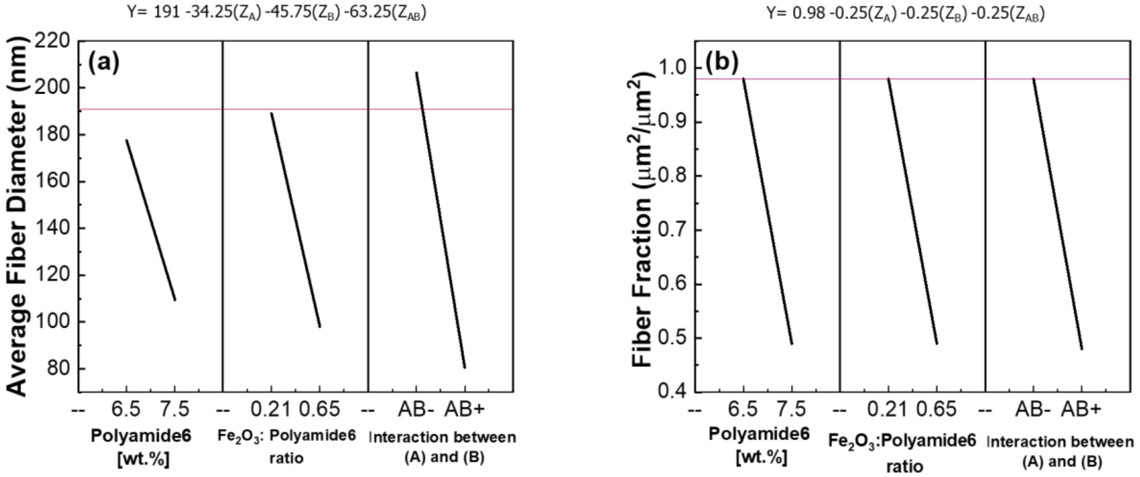

Figure S4. DOE analysis of (a) average fiber diameter and (b) fiber fraction as a function of solution compositions from second DOE.

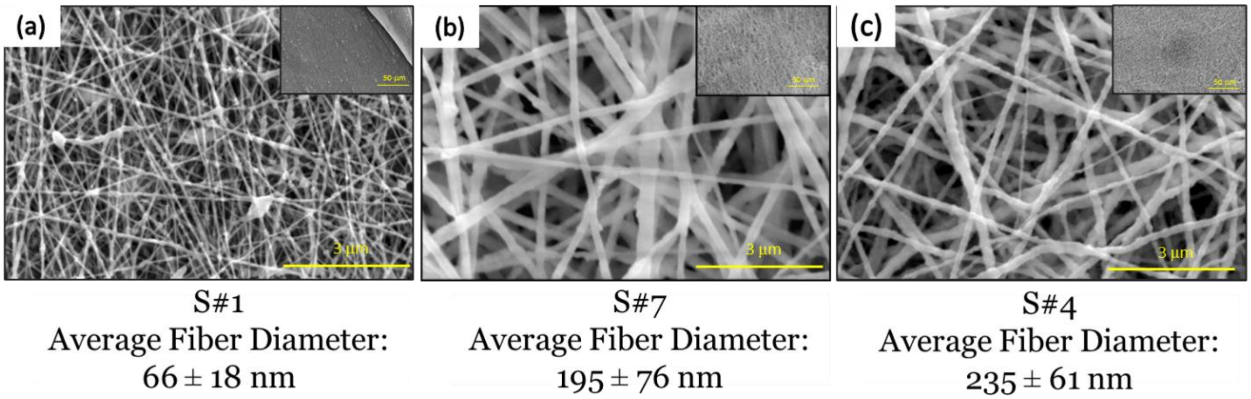

Figure S5. SEM images of polyamide 6/Fe2O3/TBAB tri-composite nanofibers with various average fiber diameters. Average fiber diameters of (a) 66 nm, (b) 195 nm and (c) 235 nm.

Table S1. BET surface area and single point adsorption total pore volume of polyamide 6/Fe_2O_3/TBAB nanofiber mats.

Sample #	Surface Area (m^2/g)	V_{tot} (cm^3/g)
1	5.21	6.88E-03
2	13.82	2.17E-02
3	10.50	1.58E-02
4	4.34	6.21E-02
5	15.84	2.75E-02
6	8.97	1.55E-02
7	9.22	1.64E-02
8	0.63	2.50E-05
9	4.27	3.45E-03

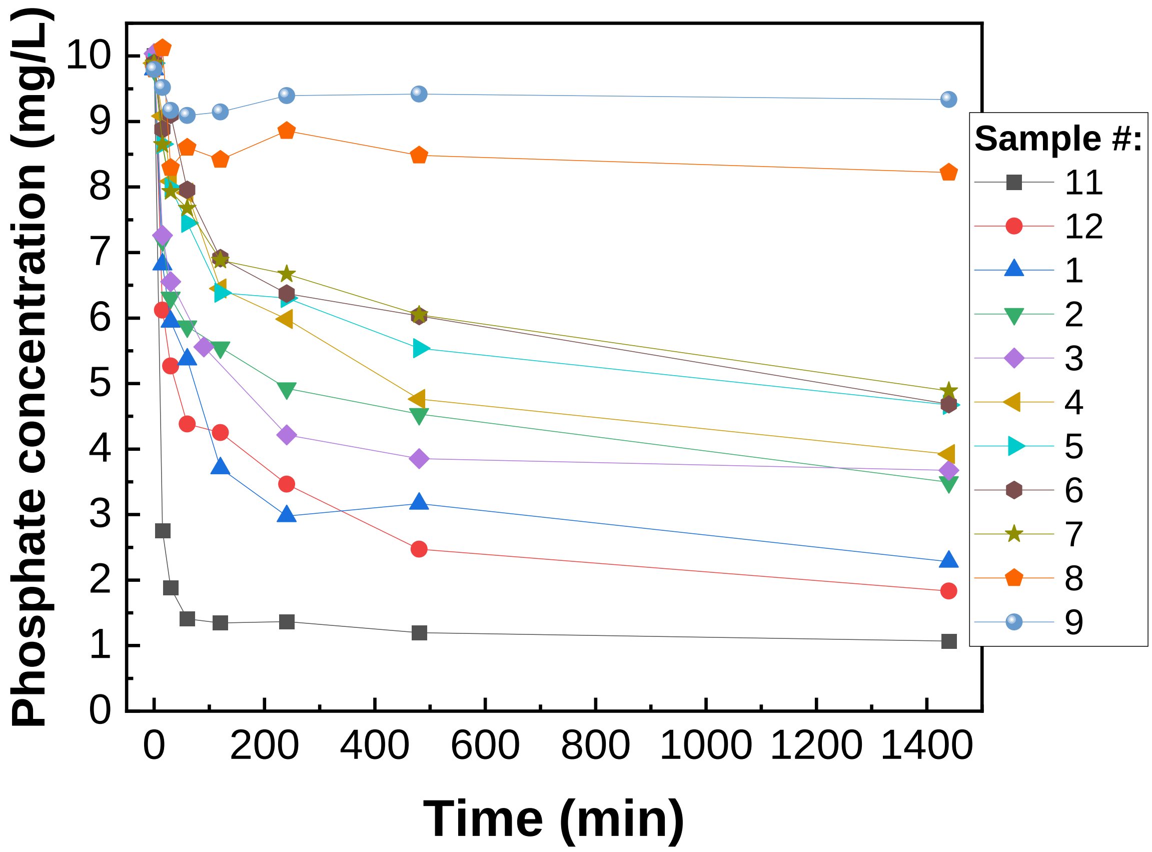

Figure S6. Effect of polyamide6/ α-Fe2O3/TBAB nanofibers on phosphate solution. Initial phosphate concentration 10mg/L, T= 23°C, absorbent dosage = 1g/L.

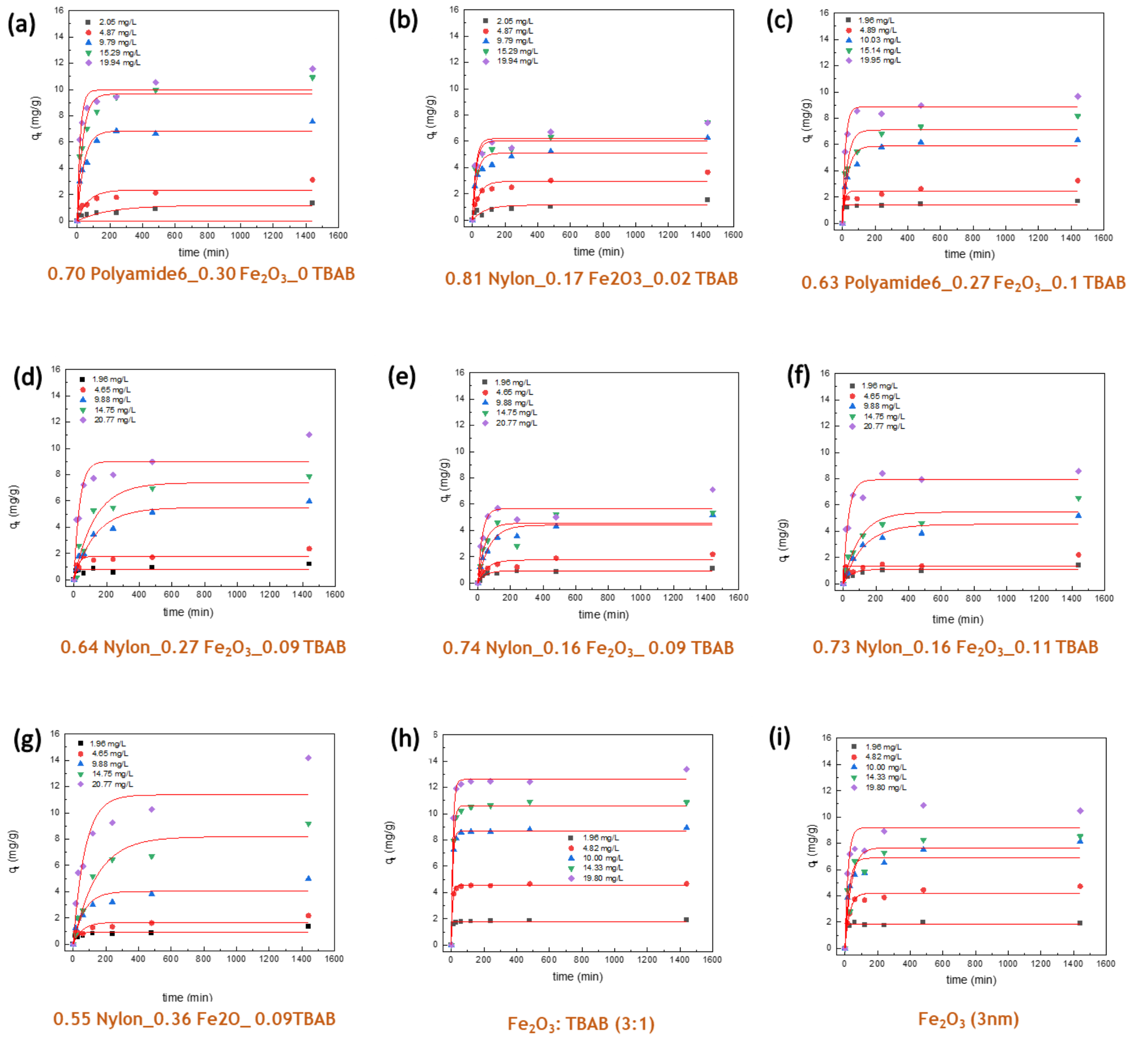

Figure S7. Effect of phosphate concentrations on the adsorption kinetics on the PA6/Fe_2O_3/TBAB composite nanofiber. Line represents fitted result with pseudo-first-order equations. (a) to (g) corresponds to Sample #1 to Sample #7 respectively and (h) to (i) corresponds to Sample #11 and Sample #12 respectively.

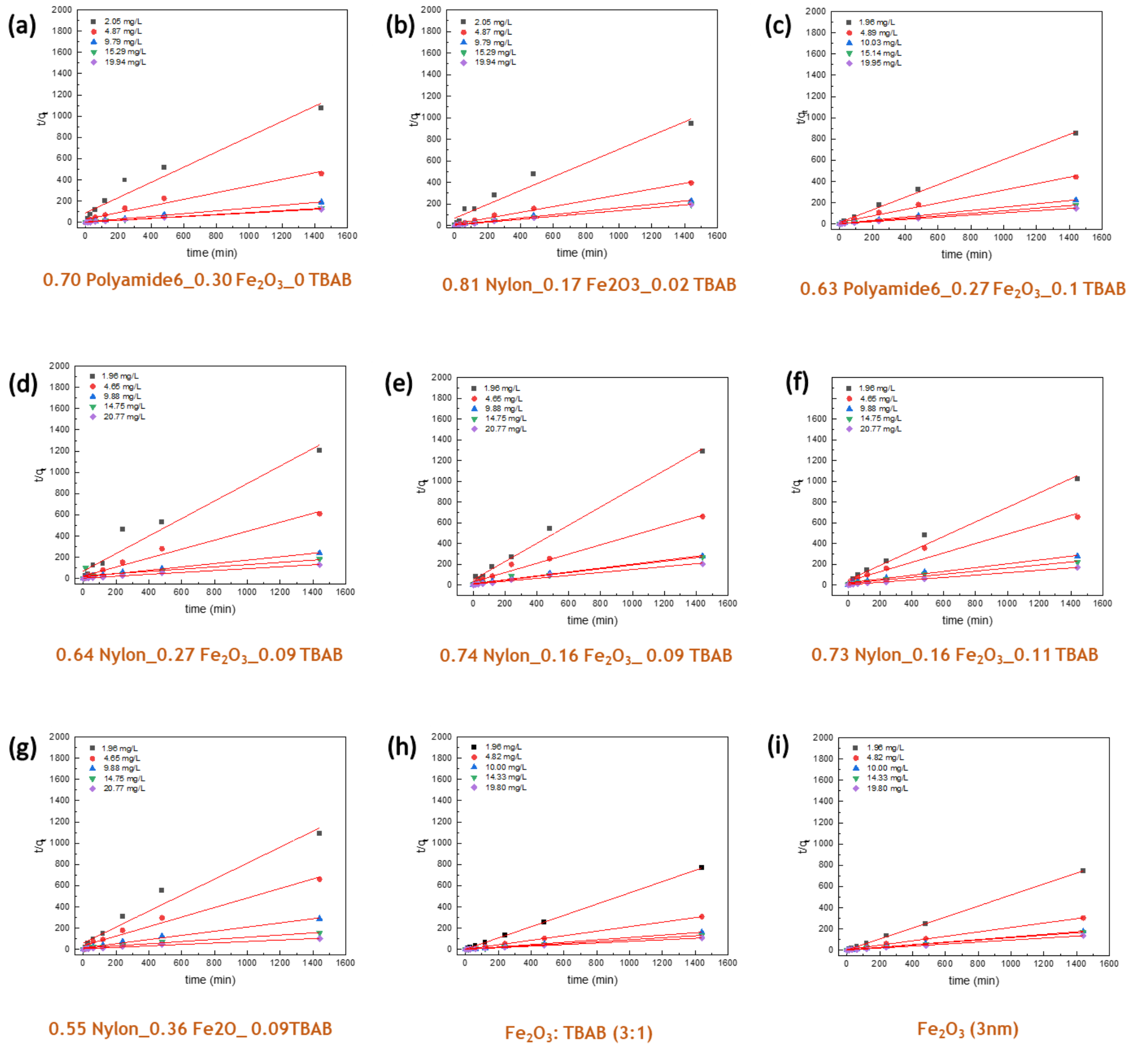

Figure S8. Effect of phosphate concentrations on the adsorption kinetics on the PA6/Fe_2O_3/TBAB composite nanofiber. Line represents fitted result with pseudo-second-order equations. (a) to (g) corresponds to Sample 1to Sample #7 respectively and (h) to (i) corresponds to Sample #11 and Sample #12 respectively.

Table S2. The parameters for the pseudo first and pseudo second order and the correlation coefficients.

	Pseudo-first order fitting			Pseudo-second order fitting		
S#	q_e (mg/g)	k_1 (min^{-1})	R^2	q_e (mg/g)	k_2 (g/(mg*min))	R^2
1	6.84	2.45E-02	0.949	7.63	4.37E-03	0.998
2	5.09	3.54E-02	0.894	6.32	3.78E-03	0.995
3	5.87	2.99E-02	0.944	6.43	7.16E-03	0.999
4	5.49	7.00E-03	0.957	6.28	2.00E-03	0.992
5	4.41	1.42E-02	0.931	5.30	3.00E-03	0.994
6	4.52	8.00E-03	0.943	2.21	1.00E-02	0.960
7	4.02	1.45E-02	0.879	5.10	2.64E-03	0.987
8	1.34	3.33E-02	0.616	1.55	3.00E-02	0.978
9	0.53	8.23E-02	0.675	0.45	3.80E-01	0.992
10	N/A	N/A	N/A	N/A	N/A	N/A
11	8.67	1.15E-01	0.997	10.90	2.89E-02	0.999
12	6.88	4.08E-02	0.904	8.24	3.86E-03	0.998

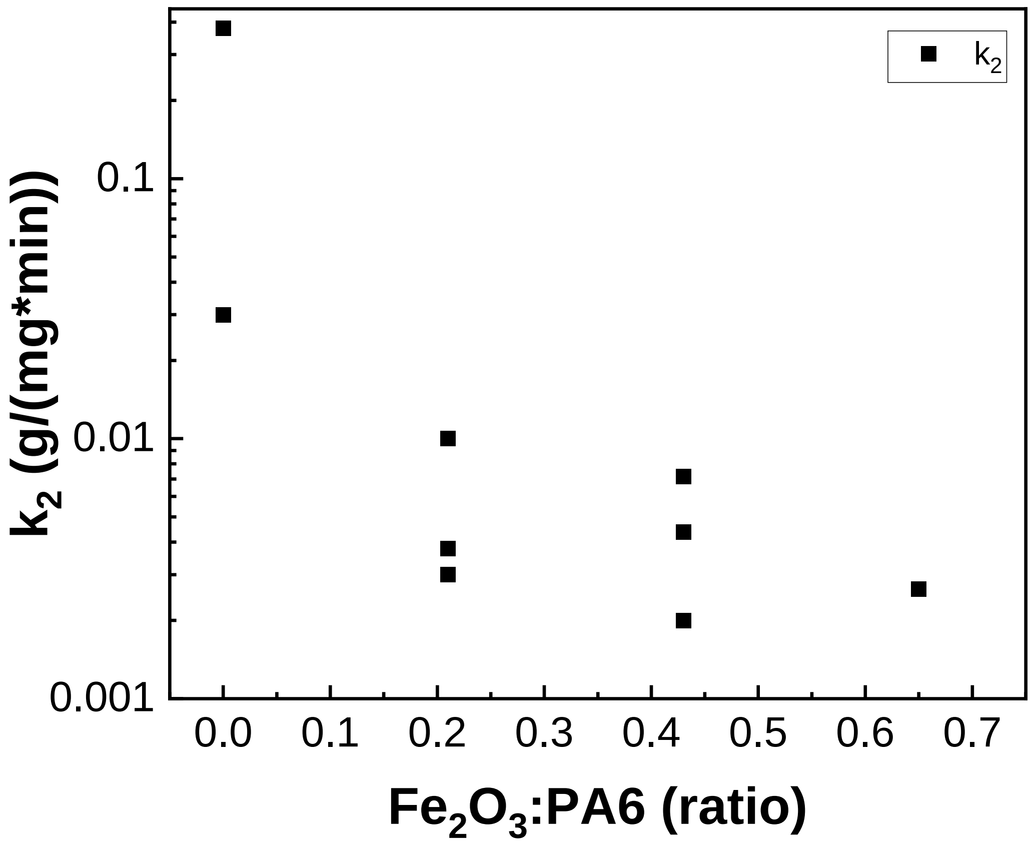

Figure S9. Pseudo second order kinetic rate constant (k2) as a function of Fe_2O_3:PA6 ratio.

Table S3. The parameters for the Langmuir and Freundlich isotherms and the correlation coefficients for the phosphate adsorption.

	Langmuir Isotherm			Freundlich Isotherm		
S#	q_{max} (mg P/ g)	K_L (L/mg)	R^2	n	k_F (mg/g)/(mg/L)^n	R^2
1	20.31	0.19	0.916	0.61	3.56	0.820
2	8.89	0.56	0.988	0.36	3.32	0.877
3	13.80	0.22	0.986	0.51	2.99	0.985
4	47.43	0.03	0.983	0.87	1.53	0.976
5	10.18	0.15	0.957	0.56	1.66	0.911
6	17.10	0.08	0.978	0.66	1.63	0.970
7	N/A	N/A	N/A	N/A	N/A	N/A
8	1.65	4.43	0.831	0.08	1.33	0.201
9	0.99	0.18	0.692	0.41	0.23	0.461
10	N/A	N/A	N/A	N/A	N/A	N/A
11	13.22	2.30	0.976	0.33	7.46	0.947
12	9.32	10.27	0.969	0.21	6.47	0.927

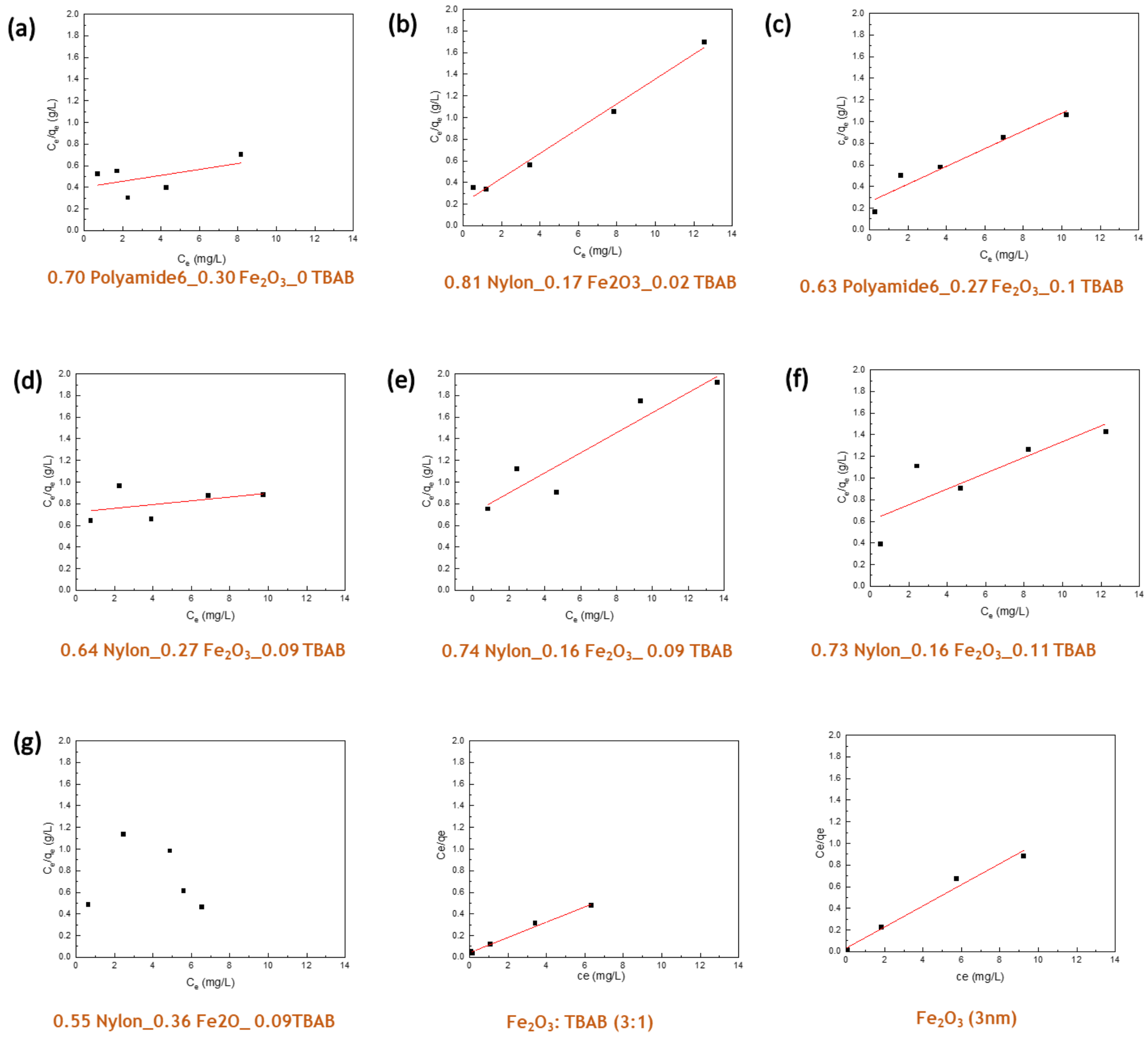

Figure S10. Langmuir adsorption isotherm for the adsorption of PA6/Fe_2O_3/TBAB nanofiber membranes. (a) to (g) corresponds to Sample #1 to Sample #7 respectively and (h) and (i) corresponds to Sample #11 and Sample #12 respectively.

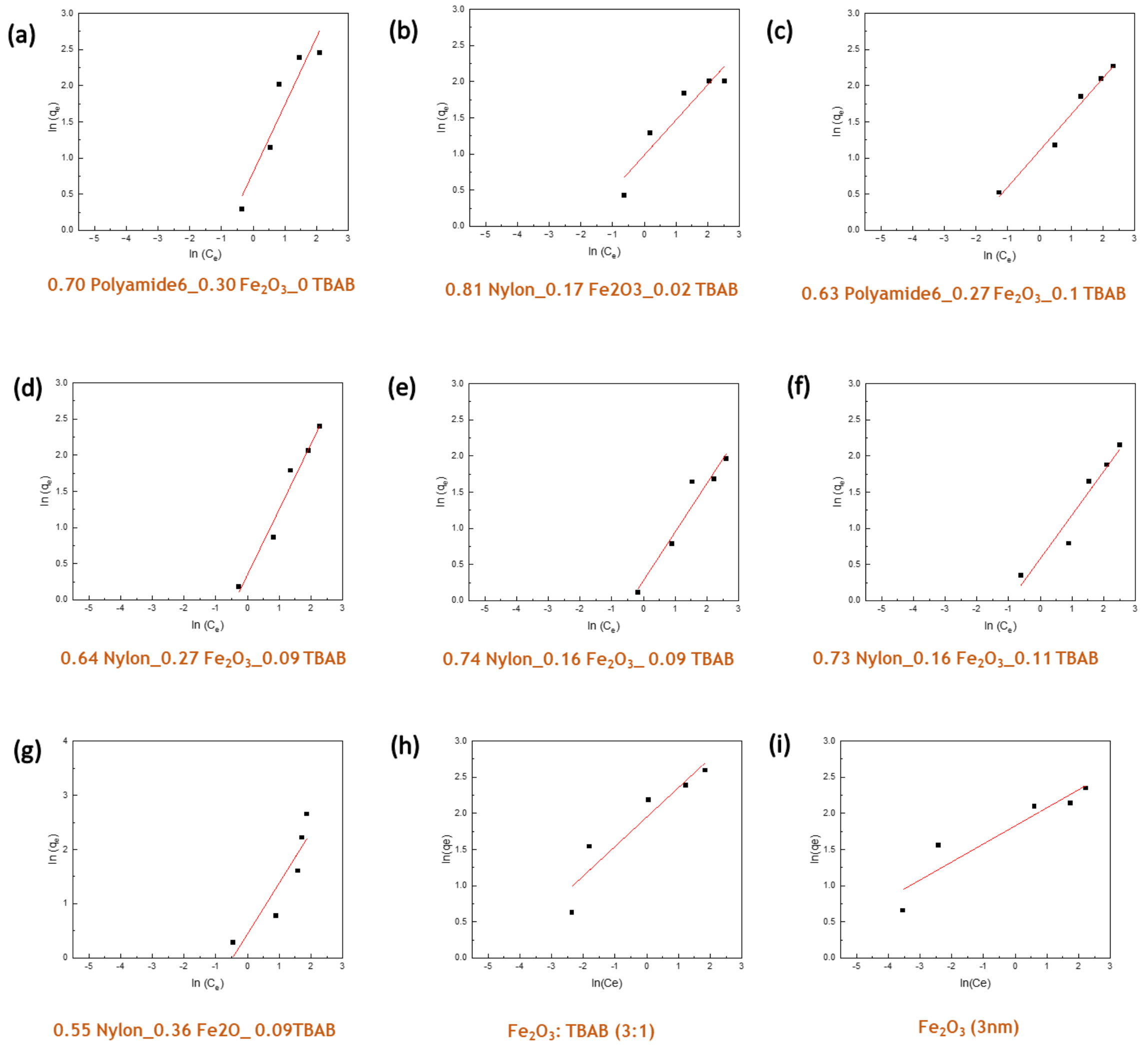

Figure S11. Freundlich adsorption isotherm for the adsorption of PA6/Fe_2O_3/TBAB nanofiber membranes. (a) to (g) corresponds to Sample #1 to Sample #7 respectively and (h) and (i) corresponds to Sample #11 and Sample #12 respectively.

Table S4. Comparative analysis of phosphate adsorption capacity with previous findings.

Material	Specific surface area (m^2/g)	Adsorption type	Adsorption capacity (mg/g)	Adsorption kinetics	Ref
Hydrous iron oxide modified diatomite (HIOMD)	80.44	Langmuir	25.02	N/A	5
Ferrihydrite	178.80	Langmuir	66.60	5.00E-05	6
Magnetite	123.10	Freundlich	57.80	2.00E-05	
Goethite	86.95	Freundlich	50.50	1.00E-05	
Granulated ferric hydroxide (GFH)	280	Langmuir	23.30	N/A	7
APANF@Fe_2O_3	N/A	Langmuir	6.34	5.00E-03	8
Granular ferric hydroxide (GFH)	N/A	Freundlich	6.54	4.00E-02	9
Alpha- goethite (Iron oxide tailing)	47.90	Langmuir-Freundlich	12.65	4.10E-01	10
Magnetite NP stabilized with sodium carboxymethyl cellulose (CMC-NP)	N/A	N/A	3.20	N/A	11
Magnetic iron oxide	82.20	Freundlich and Redlich–Peterson	5.03	N/A	12
Iron oxide immobilized onto nano-sized magnetic layer	1024	Langmuir	N/A	N/A	13
Fe_3O_4	65.78	Langmuir	11.20	N/A	14
Fe_3O_4@SiO_2	62.14	Langmuir	12	N/A	
Fe_3O_4@SiO_2@La_2O_3	47.73	Langmuir	27.80	N/A	
ZrO_2@Fe_3O_4	135.80	Langmuir	16	1.75E+00	15
ZrO_2@SiO_2@Fe_3O_4	17.10	Langmuir	6.33	2.40E-01	
PAN/Fe_2O_3/TBAB NF	N/A	Langmuir	17.05	5.30E-03	16
PA6/Fe_2O_3/TBAB NF	13.82	Langmuir	52.30	3.748E-03	This study

Table S5. Mechanical Properties of PA6/α-Fe_2O_3/TBAB composite nanofibers.

S#	Young's Modulus (Pa)	Yield Strength (Pa)	Ultimate Tensile Strength (Pa)	Toughness ($J\times m^{-3}$)	Strain to Fracture (ε)
1	2.55×10^7	1.79×10^6	2.76×10^6	6.55×10^5	3.03×10^{-1}
2	2.06×10^8	8.59×10^6	1.27×10^7	1.35×10^6	1.31×10^{-1}
3	1.26×10^8	3.86×10^6	5.13×10^6	4.71×10^5	1.95×10^{-2}
4	2.00×10^8	3.08×10^6	5.96×10^6	2.80×10^5	7.10×10^{-2}
5	1.82×10^7	8.30×10^6	$1.06E\times10^7$	9.01×10^5	1.21×10^{-1}
6	7.47×10^7	4.15×10^5	6.39×10^6	9.16×10^5	1.52×10^{-1}
7	5.72×10^7	1.71×10^5	1.94×10^6	8.09×10^4	5.13×10^{-2}
8	2.54×10^7	2.19×10^6	2.86×10^6	5.07×10^5	1.73×10^{-1}
9	1.35×10^8	1.44×10^7	1.65×10^7	2.52×10^6	2.12×10^{-1}
10	N/A	N/A	N/A	N/A	N/A

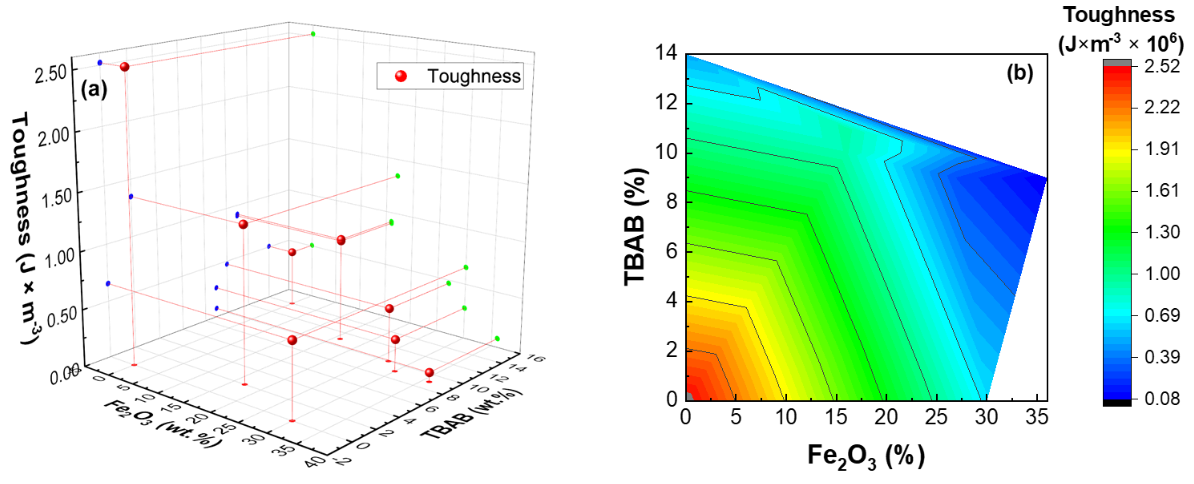

Figure S12. 3D (a) and contour (b) plots of nanofiber's toughness as function to composition.

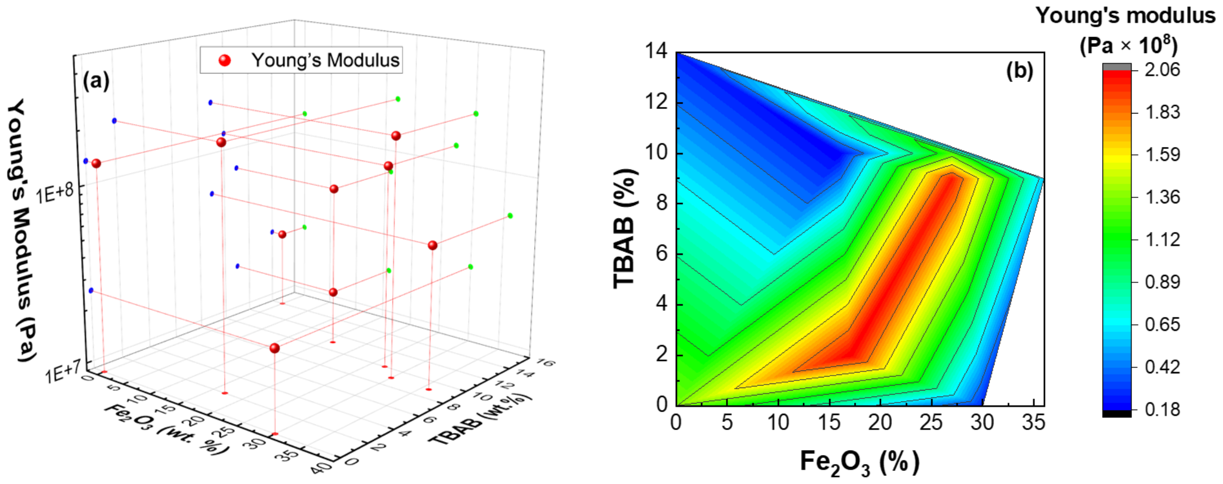

Figure S13. 3D (a) and contour (b) plots of nanofiber's Young's modulus as function to composition.

Table S6. Comparison of calculated mechanical properties of Polyamide6 and PAN tri-composite nanofibers.

Sample Composition	Young's Modulus (Pa)	Yield Strength (Pa)	Ultimate Tensile Strength (Pa)	Toughness ($J \times m^{-3}$)	Strain to Fracture (ε)
**0.63 PA6/ 0.27 Fe_2O_3/ 0.1 TBAB**	$1.26x10^8$	$3.86\ x10^6$	$5.13\ x10^6$	$4.71\ x10^5$	$1.95\ x10^{-2}$
**0.62 PAN/ 0.27 Fe_2O_3/ 0.11 TBAB**	$6.13\ x10^6$	$2.30\ x10^5$	$2.96\ x10^5$	$1.46\ x10^4$	$7.10\ x10^{-2}$
